# Development and validation of a risk prediction model for postpartum metabolic syndrome in women with gestational diabetes mellitus: A retrospective cohort study

**DOI:** 10.1097/MD.0000000000048462

**Published:** 2026-04-24

**Authors:** Fei Wang, Danyang Ye, Yifei Ma, Kai Zhao, Zhaofen Liu, Lixia Cheng

**Affiliations:** a Department of Endocrinology, The First Affiliated Hospital of Shandong Second Medical University (Weifang People’s Hospital), Weifang, Shandong, China; b Department of Neuroendocrinology, The 989th Hospital of Joint Logistics Support Force of PLA, Weifang, Shandong, China; c Department of Endocrinology, Affiliated Hospital of Qingdao University, Qingdao, Shandong, China; d Department of Hepatobiliary and Pancreatic Surgery, Second Hospital of Jilin University, Changchun, Jilin, China; e Outpatient Department, The First Affiliated Hospital of Shandong Second Medical University (Weifang People’s Hospital), Weifang, Shandong, China; f Health Management Center Department, The First Affiliated Hospital of Shandong Second Medical University (Weifang People’s Hospital), Weifang, Shandong, China.

**Keywords:** Gestational diabetes mellitus (GDM), metabolic syndrome (MetS), nomogram, prediction model

## Abstract

Women with gestational diabetes mellitus (GDM) face an elevated risk of developing metabolic syndrome (MetS) after delivery. Early identification of high-risk individuals is essential to prevent long-term metabolic and cardiovascular complications, yet predictive tools for postpartum MetS remain scarce. To develop and validate a nomogram-based model for estimating postpartum MetS risk in women with GDM using routine clinical and laboratory data. This retrospective cohort study included 522 GDM patients treated at a tertiary hospital in Weifang, China, from April 2022 to September 2024. Participants were randomly assigned to training (n = 404) and validation (n = 118) cohorts. Candidate predictors identified by univariate logistic regression were further analyzed by multivariate regression to determine independent risk factors. A nomogram was built from these predictors, and model performance was evaluated using receiver operating characteristic curves, area under the curve, calibration plots, and decision curve analysis. Robustness was tested through sensitivity and subgroup analyses. The final model included 5 independent predictors: polycystic ovary syndrome (PCOS), homeostatic model assessment for insulin resistance (HOMA-IR), interleukin-6 (IL-6), high-density lipoprotein cholesterol (HDL-C), and serum uric acid. The model achieved area under the curves of 0.818 (95% CI 0.743–0.892) and 0.957 (95% CI 0.926–0.989) in the training and validation cohorts, respectively. Calibration showed strong agreement between predicted and observed outcomes, with mean absolute errors of 0.026 and 0.082. Decision curve analysis confirmed a high clinical net benefit, and sensitivity and subgroup analyses demonstrated stable performance across clinical strata. This validated nomogram, based on readily available clinical and biochemical indicators, accurately predicts postpartum MetS risk in women with GDM and may facilitate early detection and targeted prevention in high-risk patients.

## 1. Introduction

Gestational diabetes mellitus (GDM) is one of the most common complications of pregnancy, characterized by abnormal glucose metabolism first detected during pregnancy.^[1]^ Its incidence has been rising globally, partly due to changes in lifestyle and an aging population.^[2,3]^ Recent epidemiological studies show that in China, the prevalence of GDM ranges from 14% to 20%, making it an important public health concern affecting both mothers and their children.^[4,5]^

Metabolic syndrome (MetS) is a cluster of conditions, including central obesity, insulin resistance, dyslipidemia, and hypertension. It is recognized as a major risk factor for cardiovascular disease and type 2 diabetes.^[6,7]^ A growing body of research confirms that women with GDM are at much greater risk of developing MetS after delivery, compared with women who do not experience GDM during pregnancy.^[2,8-12]^ These 2 conditions share similar metabolic disruptions, and their underlying mechanisms are closely linked.^[13]^ Early identification and intervention for GDM patients at high risk of MetS are vital for preventing long-term maternal complications and improving pregnancy outcomes.^[14]^

Among numerous biomarkers associated with MetS, several key factors have shown particular biological and predictive relevance. Interleukin-6 (IL-6), a central pro-inflammatory cytokine, contributes to insulin resistance, endothelial dysfunction, and lipid metabolism disorders, and its elevation has been observed in both GDM and MetS patients.^[15-17]^ Serum uric acid (UA) reflects oxidative stress and impaired metabolic clearance, and elevated UA levels have been independently linked to insulin resistance, dyslipidemia, and postpartum MetS risk.^[18,19]^ Homeostasis model assessment of insulin resistance (HOMA-IR) is a direct quantitative measure of insulin resistance, representing a core metabolic disturbance common to both GDM and MetS.^[20,21]^ High-density lipoprotein cholesterol (HDL-C), a key component of lipid metabolism, is inversely related to insulin resistance and inflammation; reduced HDL-C is an established feature of MetS and a predictor of cardiovascular complications.^[22,23]^ Collectively, these biomarkers capture complementary aspects of glucose-lipid metabolism, inflammatory status, and insulin sensitivity, providing a solid physiological rationale for their inclusion in the current predictive model.

While prior studies have explored risk factors associated with GDM and its complications, few quantitative prediction tools are available specifically for estimating MetS risk in this high-risk population.^[24]^ Most existing models target the general population or focus on single risk factors, lacking a comprehensive approach for GDM patients.^[25,26]^ Moreover, many published models are limited by small sample sizes, insufficient external validation, and lack of subgroup or sensitivity analyses, limiting their usefulness in real-world clinical practice.

In clinical practice, nomograms that integrate multiple clinical and laboratory parameters can provide individualized risk estimates for MetS, allowing clinicians to identify and manage high-risk patients more effectively. From a translational perspective, developing a simple and reliable risk model may enable early postpartum screening, guide timely lifestyle or pharmacologic interventions, and ultimately reduce long-term cardiovascular and metabolic complications among women after GDM.

To address this gap, we used clinical data from a large tertiary hospital in northern China to develop and validate a nomogram-based risk prediction model for postpartum MetS in GDM patients. This study systematically identified independent risk factors using logistic regression, evaluated model performance with receiver operating characteristic (ROC) and calibration curves, and further confirmed robustness through decision curve analysis, sensitivity analyses, and subgroup validation. Our goal was to offer clinicians a practical and effective tool for early identification and precision management of high-risk women after GDM.

## 2. Materials and methods

### 2.1. Study population

This was a single-center, retrospective cohort study. Data were collected from the clinical database of a tertiary hospital in Weifang, China, between April 2022 and September 2024. Eligible participants were women diagnosed with GDM by oral glucose tolerance test (OGTT) and who had received regular prenatal care with complete clinical records.

**Figure 1.**
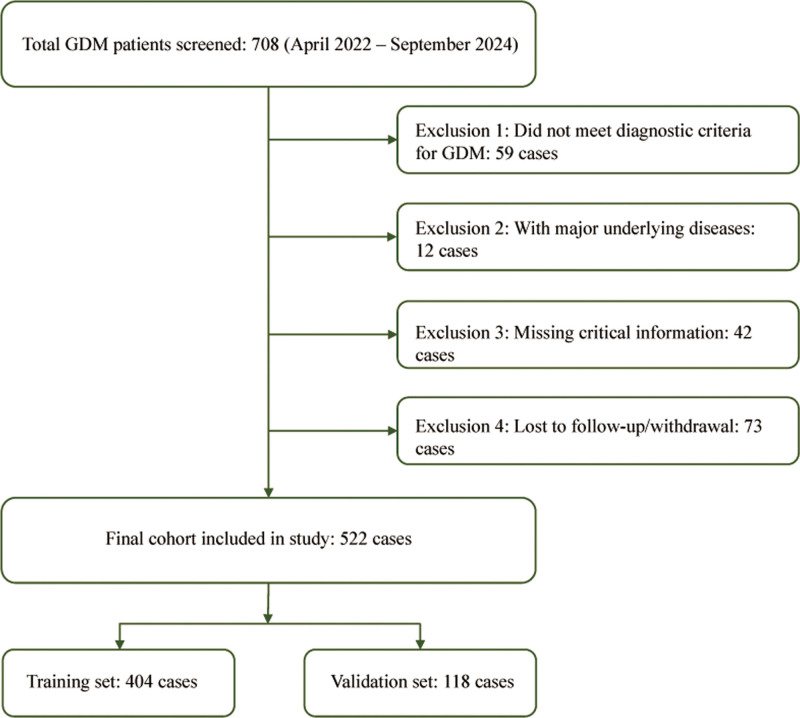
Flowchart of patient selection. Flowchart illustrating the inclusion, exclusion, and allocation of study participants into the training and validation cohorts. GDM = gestational diabetes mellitus.

Inclusion criteria: pregnant women meeting diagnostic criteria for GDM, availability of complete postpartum follow-up data, including defined MetS outcomes. Exclusion criteria: diagnosis of diabetes before pregnancy or presence of serious preexisting conditions (such as chronic kidney disease or malignancy), multiple pregnancies or pregnancy termination (miscarriage or stillbirth), missing key data or loss to follow-up, refusal to participate or consent to follow-up. Patients were randomly assigned to the training or validation cohort at a ratio of approximately 3.5:1. Of 708 patients screened, 522 met all criteria – 404 in the training cohort and 118 in the validation cohort. The patient selection flow is shown in Figure 1.

### 2.2. Data collection and variable definitions

All data were collected by trained healthcare staff during prenatal visits and delivery, and checked by designated personnel to ensure accuracy.

#### 2.2.1. Demographics and pregnancy complications

Age (years, at first prenatal visit), prepregnancy body mass index (Pre-BMI, kg/m^2^, from prepregnancy or first prenatal record), History of polycystic ovary syndrome (PCOS), resting heart rate (HR, bpm), systolic and diastolic blood pressure (SBP/DBP, mm Hg), gestational Hypertension (GestHyper), history of gestational diabetes mellitus (GDM History), family history of diabetes, glycemic control (GlyControl), asthma history, dyslipidemia during pregnancy.

#### 2.2.2. Biochemical, electrolyte, and inflammatory markers (typically collected at 24–28 weeks gestation unless otherwise noted)

Fasting blood glucose (mmol/L), OGTT 2-hour glucose (OGTT-2h, mmol/L, 26–28 weeks), glycated hemoglobin (HbA1c, %), serum uric acid (UA, µmol/L), homeostasis model assessment of insulin resistance (HOMA-IR), insulin index, total cholesterol (TC, mmol/L), low-density lipoprotein cholesterol (LDL-C, mmol/L), high-density lipoprotein cholesterol (HDL-C, mmol/L), triglycerides (TG, mmol/L), interleukin-6 (IL-6, pg/mL), hemoglobin (Hb, g/L), red cell distribution width (RDW, %), neutrophil/lymphocyte ratio (NLR), platelet/lymphocyte ratio (PLR), serum potassium (K, mmol/L), serum sodium (Na, mmol/L), serum chloride (Cl, mmol/L), serum calcium (Ca, mmol/L), serum magnesium (Mg, mmol/L), and N-terminal pro-brain natriuretic peptide (NT-proBNP, pg/mL, late pregnancy).

#### 2.2.3. Ultrasound and special tests (mainly late pregnancy, some in mid-pregnancy)

Pulmonary artery pressure (PAP, mm Hg), amniotic fluid index (cm), left ventricular ejection fraction (%), forced vital capacity (L), forced expiratory volume in 1 second (FEV_1_, L), FEV_1_/forced vital capacity ratio, and maximal voluntary ventilation (L/min).

#### 2.2.4. Delivery and postpartum outcomes

Neonatal length (cm), gestational age at delivery (weeks), and neonatal intensive care unit admission rate.

#### 2.2.5. Definition of MetS

Postpartum metabolic syndrome (MetS) was diagnosed if any 3 or more of the following were met: body mass index (BMI) ≥ 25 kg/m^2^, systolic blood pressure ≥ 130 mm Hg or diastolic blood pressure ≥ 85 mm Hg, fasting plasma glucose ≥ 5.6 mmol/L, triglycerides (TG) ≥ 1.7 mmol/L or high-density lipoprotein cholesterol (HDL-C) < 1.3 mmol/L, homeostasis model assessment of insulin resistance (HOMA-IR) ≥ 2.5, serum uric acid (UA) ≥ 357 µmol/L, and interleukin-6 (IL-6) ≥ 5 pg/mL.^[27,28]^ The timing and reference ranges for all measurements are provided in Table S1, Supplemental Digital Content, https://links.lww.com/MD/R739.

#### 2.2.6. Statistical analysis

All analyses were conducted using R version 4.4.3 (R Foundation for Statistical Computing, Vienna, Austria). Data processing: All raw data were checked for consistency, missing values, and outliers. Descriptive statistics: Continuous variables were reported as mean ± SD (if normally distributed) or median (IQR, if skewed). Group comparisons used *t*-tests or Mann–Whitney *U* tests as appropriate. Categorical variables were reported as counts (%) and compared with the chi-square or Fisher’s exact test. Standardized mean difference assessed baseline balance between training and validation sets. Variable selection and modeling: In the training cohort, each candidate predictor underwent univariate logistic regression; variables with *P* < .05 were included in multivariate analysis to identify independent predictors (OR and 95% CI reported). Stepwise methods (AIC/BIC) were used to prevent overfitting. Nomogram construction: a risk nomogram was developed using the *rms* package in R. Model evaluation: Discrimination was assessed by plotting ROC curves and calculating area under the curve (AUC) with 95% CIs for both the training and validation sets (pROC package). Calibration was evaluated by plotting bootstrap calibration curves with 1000 resamples (using the *rms* package calibrate function). Internal validation of the nomogram was further performed using the bootstrap resampling method (1000 iterations, *rms* package validate function) to calculate the concordance index (C-index) and assess model stability and potential overfitting; the bootstrap-corrected C-index was used to evaluate discrimination performance. Clinical utility was assessed through decision curve analysis at various thresholds (using rmda/stdca packages). Sensitivity and subgroup analyses were performed by stratifying key variables (such as age, Pre-BMI, and PCOS) and comparing model performance (AUC) across groups in both cohorts. All statistical tests were 2-sided, with *P* < .05 considered statistically significant. A 2-sided *P* < .05 was considered statistically significant.

## 3. Results

### 3.1. Baseline characteristics

A total of 522 GDM patients were included: 404 in the training cohort and 118 in the validation cohort. No significant differences were found in key baseline characteristics – such as age, Pre-BMI, PCOS, HOMA-IR, IL-6, HDL-C, and UA – between the 2 groups (all *P* > .05). This indicates good comparability. See Table 1 for details.

**Table 1:** Baseline characteristics of the training and validation cohorts.

Categorical variables						
Variable	Total (n = 522)	Train (n = 404)	Validation (n = 118)	*P* value	Test	SMD
MetS (%)				.102	Fisher	0.086
No	444 (85.1%)	355 (87.9%)	102 (86.4%)			
Yes	78 (14.9%)	49 (12.1%)	16 (13.6%)			
Gestational hypertension (%)				.056	Chi-square	0.209
No	333 (63.8%)	267 (66.1%)	66 (55.9%)			
Yes	189 (36.2%)	137 (33.9%)	52 (44.1%)			
Glycemic Control (%)				.879	Chi-square	0.023
No	450 (86.2%)	349 (86.4%)	101 (85.6%)			
Yes	72 (13.8%)	55 (13.6%)	17 (14.4%)			
Asthma (%)				.357	Chi-square	0.099
No	452 (86.6%)	353 (87.4%)	99 (83.9%)			
Yes	70 (13.4%)	51 (12.6%)	19 (16.1%)			
Dyslipidemia (%)				.345	Fisher	0.174
No	516 (98.9%)	398 (98.5%)	118 (100.0%)			
Yes	6 (1.1%)	6 (1.5%)	0 (0.0%)			
Family history of diabetes (%)				.190	Chi-square	0.182
No	470 (95.8%)	364 (90.1%)	106 (89.8%)			
Yes	52 (10.0%)	40 (9.9%)	12 (10.2%)			
Gestational weeks (%)				.593	Chi-square	0.059
≥37	317 (60.7%)	248 (61.4%)	69 (58.5%)			
<37	205 (39.3%)	156 (38.6%)	49 (41.5%)			
NICU admission (%)				.753	Chi-square	0.040
No	275 (52.7%)	211 (52.2%)	64 (54.2%)			
Yes	247 (47.3%)	193 (47.8%)	54 (45.8%)			
PCOS (%)				.114	Chi-square	0.085
No	475 (91.0%)	379 (93.8%)	109 (92.4%)			
Yes	47 (9.0%)	25 (6.2%)	9 (7.6%)			

Continuous variables						
Variable	Total (Mean ± SD)	Train (Mean ± SD)	Validation (Mean ± SD)	*P* value	Test	SMD
Age (yr)	29.19 (5.16)	29.52 (5.33)	28.75 (5.48)	.234	*t*-test	0.017
Pre-BMI (kg/m^2^)	27.13 (5.67)	27.80 (5.88)	26.83 (5.41)	.323	*t*-test	0.145
HR (bpm)	70.59 (8.77)	70.18 (8.24)	72.02 (10.31)	.045	*t*-test	0.167
SBP (mm Hg)	135.57 (15.59)	135.73 (15.66)	135.03 (15.43)	.666	*t*-test	0.045
DBP (mm Hg)	76.98 (9.78)	76.74 (9.70)	77.78 (10.07)	.311	*t*-test	0.105
GDM history	0.06 (0.24)	0.05 (0.23)	0.08 (0.28)	.228	*t*-test	0.119
Insulin index	2.02 (0.85)	1.96 (0.83)	2.20 (0.91)	.007	*t*-test	0.276
NLR	4.19 (1.06)	4.13 (0.94)	4.38 (1.38)	.025	*t*-test	0.209
PLR	157.50 (55.73)	157.04 (55.24)	159.10 (57.58)	.724	*t*-test	0.037
Hb (g/L)	138.33 (14.47)	138.39 (14.54)	138.10 (14.32)	.849	*t*-test	0.020
RDW (%)	12.92 (1.09)	12.93 (1.16)	12.86 (0.80)	.502	*t*-test	0.077
FBG (mmol/L)	5.39 (0.98)	5.31 (0.82)	5.48 (1.06)	.044	*t*-test	0.131
K (mmol/L)	4.20 (0.37)	4.21 (0.37)	4.18 (0.38)	.463	*t*-test	0.076
Na (mmol/L)	141.67 (2.07)	141.65 (2.10)	141.73 (1.99)	.699	*t*-test	0.041
Cl (mmol/L)	104.75 (2.26)	104.78 (2.26)	104.68 (2.26)	.692	*t*-test	0.042
Ca (mmol/L)	2.30 (0.11)	2.31 (0.11)	2.29 (0.11)	.068	*t*-test	0.192
Mg (mmol/L)	0.93 (0.07)	0.93 (0.07)	0.92 (0.07)	.049	*t*-test	0.106
IL-6 (pg/mL)	7.47 (3.89)	7.49 (4.03)	7.44 (3.95)	.101	*t*-test	0.051
EF (%)	61.97 (2.52)	61.95 (2.33)	62.03 (3.09)	.752	*t*-test	0.031
TC (mmol/L)	3.51 (0.42)	3.50 (0.42)	3.54 (0.41)	.342	*t*-test	0.100
LDL (mmol/L)	4.41 (0.69)	4.34 (0.58)	4.47 (0.63)	.051	*t*-test	0.122
TG (mmol/L)	2.88 (0.34)	2.88 (0.34)	2.87 (0.34)	.629	*t*-test	0.050
HDL-C (mmol/L)	1.71 (0.50)	1.80 (0.46)	1.79 (0.51)	.701	*t*-test	0.003
AFI (cm)	0.94 (0.09)	0.94 (0.09)	0.95 (0.09)	.331	*t*-test	0.101
PAP (mm Hg)	26.65 (4.10)	26.61 (4.26)	26.76 (3.51)	.725	*t*-test	0.039
FVC (L)	112.93 (22.17)	113.56 (22.61)	110.79 (20.55)	.233	*t*-test	0.128
FEV1 (L)	111.00 (25.80)	111.36 (26.22)	109.77 (24.36)	.557	*t*-test	0.063
FEV1/FVC (%)	79.59 (10.84)	79.31 (10.43)	80.55 (12.13)	.272	*t*-test	0.110
MVV (L/min)	89.26 (22.90)	89.38 (23.59)	88.87 (20.48)	.831	*t*-test	0.023
OGTT-2h (mmol/L)	1.92 (0.33)	1.91 (0.34)	1.93 (0.28)	.536	*t*-test	0.068
Neonatal Length (cm)	5.93 (1.35)	5.98 (1.34)	5.76 (1.39)	.133	*t*-test	0.156
UA (µmol/L)	307.10 (44.06)	302.87 (41.94)	311.56 (46.22)	.061	*t*-test	0.037
HOMA-IR	3.58 (1.05)	3.60 (0.97)	3.55 (1.13)	.101	*t*-test	0.107
HbA1c (%)	6.03 (0.57)	6.00 (0.55)	6.12 (0.60)	.050	*t*-test	0.201
NT-proBNP (pg/mL)	122.28 (42.70)	122.70 (42.84)	120.87 (42.34)	.684	*t*-test	0.043

Comparison of categorical variables (% frequency) and continuous variables (mean ± SD) between the training (n = 404) and validation (n = 118) cohorts. The 2 cohorts were generally comparable in demographic, clinical, and biochemical characteristics. A statistically significant difference was observed in the insulin index (*P* = .007), which was considered clinically acceptable and did not affect the subsequent model development or validation.

AFI = amniotic fluid index, BMI = body mass index, Ca = calcium, Cl = chloride, DBP = diastolic blood pressure, EF = ejection fraction, FBG = fasting blood glucose, FVC = forced vital capacity, FEV1 = forced expiratory volume in 1 second, GDM = gestational diabetes mellitus, Hb = hemoglobin, HbA1C = glycated hemoglobin, HDL-C = high-density lipoprotein cholesterol, HOMA-IR = homeostatic model assessment for insulin resistance, HR = heart rate, LDL = low-density lipoprotein cholesterol, IL-6 = interleukin-6, K = potassium, MetS = metabolic syndrome, Mg = magnesium, MVV = maximal voluntary ventilation, Na = sodium, NLR = neutrophil-to-lymphocyte ratio, NT-proBNP = N-terminal pro-brain natriuretic peptide, OGTT-2h = 2-hour oral glucose tolerance test, PAP= pulmonary artery pressure, PCOS = polycystic ovary syndrome, PLR = platelet-to-lymphocyte ratio, RDW = red cell distribution width, SD = standard deviation, SBP = systolic blood pressure, TC = total cholesterol, TG = triglycerides, UA = uric acid.

### 3.2. Univariate and multivariate logistic regression

Univariate logistic regression in the training cohort identified PCOS, HOMA-IR, FEV1, HDL-C, NLR, IL-6, HR, PAP, TC, and UA as significant predictors of postpartum MetS (all *P* < .05; Table 2). Multivariate logistic regression further confirmed PCOS, HOMA-IR, IL-6, HDL-C, and UA as independent predictors (Table 3). Other variables identified as significant in univariate analysis, such as FEV1, NLR, HR, PAP, and TC, were not retained in the final model due to loss of statistical significance after adjusting for confounders and potential collinearity.

**Table 2:** Univariate logistic regression analysis of predictors for postpartum MetS.

Variable	Estimate	Std. error	Statistic	*P* value	OR	2.5%	97.5%
PCOS	2.925	0.455	6.433	.000	18.640	7.644	45.450
HOMA-IR	0.698	0.143	4.899	.000	2.011	1.520	2.659
FEV1	-0.066	0.019	-3.415	.001	0.936	0.902	0.972
HDL-C	-0.848	0.288	-2.948	.003	0.428	0.244	0.752
NLR	0.384	0.138	2.775	.006	1.469	1.119	1.926
IL-6	0.005	0.002	2.606	.009	1.005	1.001	1.010
HR	0.048	0.019	2.553	.011	1.050	1.011	1.089
PAP	0.081	0.032	2.503	.012	1.084	1.018	1.154
TC	0.720	0.344	2.091	.037	2.055	1.046	4.035
UA	0.007	0.003	2.087	.037	1.007	1.000	1.014
Gest weeks	0.577	0.306	1.885	.059	1.781	0.977	3.246
Family history of diabetes	0.946	0.541	1.750	.080	2.576	0.893	7.433
FVC	0.011	0.007	1.635	.102	1.011	0.998	1.024
TG	-0.637	0.449	-1.418	.156	0.529	0.219	1.276
GestHyper	0.435	0.310	1.404	.160	1.545	0.842	2.837
NT-proBNP	0.005	0.004	1.316	.188	1.005	0.998	1.012
Asthma	0.517	0.404	1.280	.201	1.677	0.760	3.701
Neonatal length	0.154	0.120	1.278	.201	1.166	0.921	1.476
Pre-BMI	0.028	0.023	1.256	.209	1.029	0.984	1.075
PLR	0.003	0.002	1.187	.235	1.003	0.998	1.008
Ca	-1.507	1.362	-1.107	.268	0.221	0.015	3.198
RDW	0.122	0.118	1.038	.300	1.130	0.897	1.423
K	0.401	0.420	0.954	.340	1.493	0.655	3.400
Mg	-1.986	2.228	-0.891	.373	0.137	0.002	10.816
AFI	1.362	1.661	0.820	.412	3.904	0.150	101.328
LDL	0.219	0.295	0.743	.458	1.245	0.699	2.218
Age	0.020	0.029	0.705	.481	1.021	0.964	1.080
FEV1	0.004	0.006	0.665	.506	1.004	0.993	1.015
FBG	0.119	0.180	0.659	.510	1.126	0.791	1.604
GlyControl	0.246	0.417	0.589	.556	1.279	0.565	2.896
DBP	-0.009	0.016	-0.588	.557	0.991	0.961	1.022
MVV	-0.003	0.007	-0.505	.614	0.997	0.984	1.010
Na	0.037	0.075	0.489	.625	1.037	0.896	1.201
Dyslipidemia	0.377	1.106	0.341	.733	1.458	0.167	12.749
Hb	-0.003	0.011	-0.296	.768	0.997	0.977	1.018
SBP	0.003	0.010	0.285	.776	1.003	0.984	1.022
EF	-0.016	0.067	-0.234	.815	0.984	0.864	1.122
GDM history	0.143	0.641	0.223	.824	1.153	0.328	4.050
HbA1c	0.061	0.275	0.221	.825	1.063	0.620	1.822
OGTT-2h	0.074	0.454	0.164	.870	1.077	0.442	2.624
NICU admission	-0.038	0.305	-0.125	.901	0.963	0.529	1.751
Cl	0.005	0.068	0.068	.946	1.005	0.880	1.147
Insulin index	0.225	0.131	1.719	.951	1.252	0.970	1.617

Univariate logistic regression identifying variables associated with postpartum metabolic syndrome (MetS) in women with gestational diabetes mellitus (GDM).

AFI = amniotic fluid index, Ca = calcium, Cl = chloride, DBP = diastolic blood pressure, EF = ejection fraction, FEV1 = forced expiratory volume in 1 second, FVC = forced vital capacity, GDM = gestational diabetes mellitus, Hb = hemoglobin, HbA1c = glycated hemoglobin, HDL-C = high-density lipoprotein cholesterol, HOMA-IR = homeostatic model assessment for insulin resistance, HR = heart rate, IL-6 = interleukin-6, K = potassium, LDL = low-density lipoprotein cholesterol, MetS = metabolic syndrome, Mg = magnesium, MVV = maximal voluntary ventilation, Na = sodium, NICU = neonatal intensive care unit, NLR = neutrophil-to-lymphocyte ratio, NT-proBNP = N-terminal pro-brain natriuretic peptide, OGTT-2h = 2-hour oral glucose tolerance test, PAP = pulmonary artery pressure, PCOS = polycystic ovary syndrome, PLR = platelet-to-lymphocyte ratio, Pre-BMI = prepregnancy body mass index, RDW = red cell distribution width, SBP = systolic blood pressure, TC = total cholesterol, TG = triglycerides, UA = uric acid.

**Table 3:** Multivariate logistic regression analysis identifying independent predictors of postpartum MetS.

Variable	Estimate	Std.error	Statistic	*P* value	OR	2.5%	97.5%
PCOS	2.901	0.549	5.287	.000	18.197	6.206	53.351
HOMA-IR	0.728	0.169	4.322	.000	2.072	1.489	2.883
HDL-C	-1.095	0.372	-2.943	.003	0.335	0.161	0.694
UA	0.010	0.004	2.302	.021	1.010	1.001	1.018
IL-6	0.005	0.003	2.054	.040	1.005	1.000	1.010
TC	0.858	0.431	1.993	.046	2.359	1.014	5.487
PAP	0.077	0.041	1.880	.060	1.080	0.997	1.171
NLR	0.376	0.209	1.795	.073	1.456	0.966	2.196
HR	0.040	0.023	1.781	.075	1.041	0.996	1.088
FEV1	0.011	0.007	1.577	.115	1.011	0.997	1.024

Multivariate logistic regression revealed that PCOS, HOMA-IR, IL-6, HDL-C, and UA were independent predictors of postpartum MetS risk in women with GDM. These factors were incorporated into the final nomogram model for risk estimation.

FEV1 = forced expiratory volume in 1 second, GDM = gestational diabetes mellitus, HDL-C = high-density lipoprotein cholesterol, HOMA-IR = homeostatic model assessment for insulin resistance, HR = heart rate, IL-6 = interleukin-6, MetS = metabolic syndrome, NLR = neutrophil-to-lymphocyte ratio, PAP = pulmonary artery pressure, PCOS = polycystic ovary syndrome, TC = total cholesterol, UA = uric acid.

### 3.3. Nomogram construction and model performance

**Figure 2.**
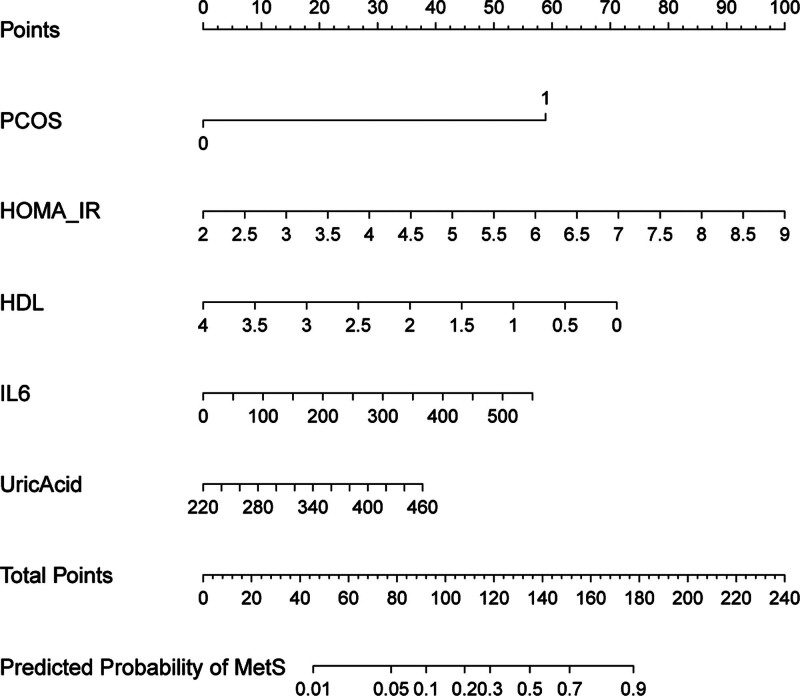
Nomogram for predicting postpartum metabolic syndrome. Nomogram-based on 5 predictors (PCOS, HOMA-IR, IL-6, HDL-C, UA) for estimating individual risk of postpartum MetS in women with GDM. GDM = gestational diabetes mellitus, HDL-C = high-density lipoprotein cholesterol, HOMA-IR = homeostatic model assessment for insulin resistance, IL-6 = interleukin-6, MetS = metabolic syndrome, UA = uric acid.

**Figure 3.**
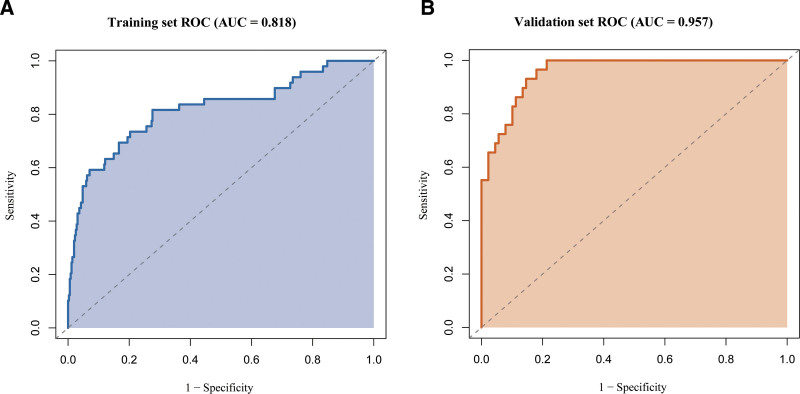
ROC curves of the prediction model. (A) ROC curve in the training cohort (AUC = 0.818, 95% CI: 0.743–0.892, *P* < .001); (B) ROC curve in the validation cohort (AUC = 0.957, 95% CI: 0.926–0.989, *P* < .001). The model demonstrates strong and statistically significant discrimination in both datasets. AUC = area under the curve, CI = confidence interval, ROC = receiver operating characteristic.

**Figure 4.**
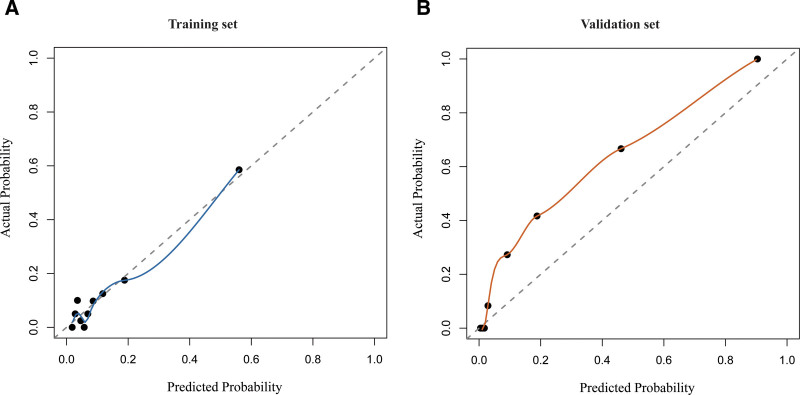
Calibration curves of the prediction model. (A) Calibration curve for the training cohort (*P* = .742 for Hosmer–Lemeshow goodness-of-fit test); (B) for the validation cohort (*P* = .681), showing good agreement between predicted and observed risks.

**Figure 5.**
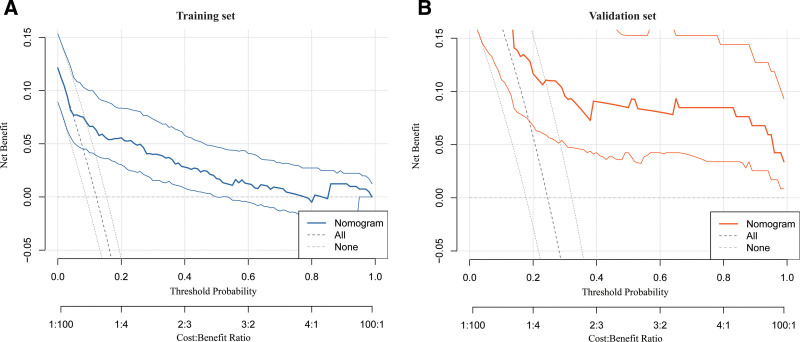
Decision curve analysis (DCA). (A) DCA for the training cohort; (B) for the validation cohort. Both curves indicate consistent clinical net benefit across various threshold probabilities (*P* < .05), demonstrating that the model provides significant added value for clinical decision-making. DCA = decision curve analysis.

A nomogram was built based on these predictors (Fig. 2). The model achieved an AUC of 0.818 (95% CI: 0.743–0.892) in the training set and 0.957 (95% CI: 0.926–0.989) in the validation set (Fig. 3), indicating strong discrimination. Internal validation using the bootstrap resampling method (1000 iterations) produced a concordance index (C-index) of 0.81 (95% CI, 0.74–0.88), confirming strong internal discrimination and suggesting that the model’s performance was not substantially overestimated. Calibration curves showed a mean absolute error of 0.026 in the training set and 0.082 in the validation set (Fig. 4), demonstrating good agreement between predicted and observed risk. Decision curve analysis revealed high net clinical benefit across a wide range of risk thresholds (Fig. 5), particularly within the clinically recommended threshold probability range of 10% to 30%, indicating good clinical utility. Detailed distributions of key predictors and predicted probabilities are provided for both cohorts, indicating that the data were representative and free of extreme values.

### 3.4. Sensitivity analysis

Stratified sensitivity analysis of PCOS, HOMA-IR, HDL-C, UA, and IL-6 showed that the model’s AUC was generally above 0.70 in all subgroups, with most exceeding 0.80 to 0.90, confirming the robustness and applicability of the model. In the validation set, for example, the AUC was 0.904 for the low HDL-C group and 0.864 for the high HDL-C group; 0.844 for those without PCOS and 0.802 for those with PCOS. The AUC was slightly lower in the low HOMA-IR group (0.655), but still above 0.7 in the high HOMA-IR group. Full results are presented in Table 4.

**Table 4:** Sensitivity analysis of the main predictors in the MetS risk prediction model.

Dataset	Sensitivity variable	Group	Sample size	AUC
Training	PCOS	No	379	0.751
Training	PCOS	Yes	25	0.729
Validation	PCOS	No	83	0.844
Validation	PCOS	Yes	35	0.802
Training	HOMA-IR	low	229	0.783
Training	HOMA-IR	high	175	0.873
Validation	HOMA-IR	low	81	0.655
Validation	HOMA-IR	high	37	0.705
Training	HDL-C	low	335	0.825
Training	HDL-C	high	69	0.751
Validation	HDL-C	low	87	0.904
Validation	HDL-C	high	31	0.864
Training	UA	low	202	0.803
Training	UA	high	202	0.819
Validation	UA	low	56	0.738
Validation	UA	high	62	0.862
Training	IL-6	low	202	0.832
Training	IL-6	high	202	0.783
Validation	IL-6	low	118	0.835

Model discrimination was evaluated by area under the curve (AUC) across subgroups stratified by PCOS, HOMA-IR, IL-6, HDL-C, and UA levels in the training and validation cohorts. The results demonstrated robust and consistent model performance (AUC > 0.80) across all subgroups.

AUC = area under the curve, HDL-C = high-density lipoprotein cholesterol, HOMA-IR = homeostatic model assessment for insulin resistance, IL-6 = interleukin-6, PCOS = polycystic ovary syndrome, UA = uric acid.

### 3.5. Subgroup analysis

Model performance was also strong across different clinical subgroups – such as age (<35 or ≥35 years), PCOS status, and Pre-BMI (<24 or ≥24). In these groups, AUCs were all above 0.79, with some as high as 0.99, further supporting the model’s broad applicability. For example, in the validation cohort, the AUC was 0.835 for age≥35, 0.804 for age <35, 0.877 for BMI < 24, and 0.854 for BMI ≥ 24. See Table 5 for full subgroup results.

**Table 5:** Subgroup analysis according to key clinical characteristics.

Dataset	Subgroup variable	Group	Sample size	AUC
Training	PCOS	No	379	0.751
Training	PCOS	Yes	25	0.729
Validation	PCOS	No	109	0.844
Validation	PCOS	Yes	9	0.767
Training	Age	≥35 yr	89	0.835
Training	Age	<35 yr	315	0.813
Validation	Age	≥35 yr	26	0.835
Validation	Age	<35 yr	92	0.804
Training	Pre-BMI	<24 kg/m^2^	76	0.913
Training	Pre-BMI	≥24 kg/m^2^	328	0.880
Validation	Pre-BMI	<24 kg/m^2^	24	0.877
Validation	Pre-BMI	≥24 kg/m^2^	94	0.854

Subgroup analysis of model discrimination (AUC) based on major clinical characteristics, including age (<35 vs ≥ 35 yr), prepregnancy body mass index (BMI < 24 vs ≥ 24 kg/m^2^), and PCOS status, in both training and validation cohorts. The model maintained stable predictive ability across different clinical strata.

AUC = area under the curve, BMI = body mass index, PCOS = polycystic ovary syndrome.

## 4. Discussion

Although previous studies have shown an increased risk of MetS among women with GDM,^[8,29,30]^ specific predictive tools for this high-risk group remain scarce, limiting evidence-based strategies for early screening and intervention. Therefore, developing a simple and effective prediction model is of great significance for guiding clinical management. This study, based on clinical data of the East Asian population, systematically constructed and validated a prediction model for the risk of MetS in GDM patients after delivery. The results showed that HDL-C, UA, HOMA-IR, IL-6, and PCOS as independent risk factors for MetS.^[31-33]^ The nomogram model constructed based on these factors showed good predictive ability and calibration in both the training set and validation set. This result provides important theoretical basis and practical tools for early identification of high-risk populations and implementation of personalized intervention in clinical practice.

Unlike previous studies that mainly focused on the general population or single-factor prediction of MetS, our study targeted a specific high-risk group – women with GDM. We comprehensively evaluated multiple pathological mechanisms, including metabolic, inflammatory, and endocrine factors.^[34]^ Previous research has shown that insulin resistance in women with GDM often persists and may worsen after delivery, which is an important mechanism underlying the development of MetS,^[35-37]^ Our findings further confirmed the strong predictive value of insulin resistance, measured by HOMA-IR, for MetS, consistent with results from several large-scale international studies.^[38,39]^ In addition, this study highlighted the important roles of the inflammatory marker IL-6 and the endocrine disorder PCOS in the development of MetS among GDM patients. It should be noted that IL-6 and UA were incorporated into the model as predictive markers rather than diagnostic tools. Their inclusion reflects the underlying inflammatory and metabolic disturbances linking GDM and MetS, which enhances the biological relevance and interpretability of the model. These indicators are not intended to replace standard diagnostic criteria for GDM but may serve as useful adjuncts for risk assessment and early identification of women at high risk of postpartum MetS. IL-6 is a key inflammatory cytokine, and its persistent mild elevation can induce insulin resistance and accelerate metabolic dysfunction,^[40,41]^ PCOS, through hormonal imbalance, further aggravates both insulin resistance and systemic inflammation, significantly increasing the risk of MetS.^[42,43]^ Although previous studies have examined IL-6 or PCOS individually in the general population, our study is the first to simultaneously include these factors in a predictive model for postpartum MetS among women with GDM. This approach makes the model more sensitive and specific in identifying high-risk individuals.

This study further discussed why only PCOS, HOMA-IR, IL-6, HDL-C, and UA became independent predictors of MetS. These factors represent the core pathological characteristics of MetS in GDM patients.^[44]^ Pre-BMI and HOMA-IR reflect the core mechanisms of insulin resistance and abnormal metabolism of adipose tissue,^[45-47]^ while HDL-C, as an important indicator of lipid metabolism, is closely related to key pathological links of metabolic syndrome such as atherosclerosis.^[48]^ UA is one of the markers of oxidative stress and inflammatory response, and is closely related to metabolic disorders and insulin resistance.^[49]^ Therefore, the combined action of these factors reflects the pathological basis of MetS occurrence in GDM patients, which is consistent with the findings of previous studies,^[8,50,51]^ further verifying the rationality of the results of this study.

In the sensitivity analysis, IL-6 in the validation set was represented only by the low-level group due to the limited number of patients with high IL-6 levels. This reflects a common data imbalance observed in real-world clinical studies and suggests the need for future analyses with larger sample sizes to verify the findings. The decision curve analysis showed that the model provided a high clinical net benefit across a wide range of threshold probabilities, indicating that clinicians can flexibly determine intervention thresholds for personalized management. However, the optimal threshold should still be determined according to real-world clinical contexts and resource availability. Previous literature recommends using a threshold range of 10% to 30% (0.1–0.3) for most clinical decision-making.^[52]^ While the decision curve analysis in this study performed well within this range, it also demonstrated a high net benefit beyond these limits, suggesting that the model offers flexibility and broad applicability in clinical use. The calibration analysis indicated good agreement between predicted and observed outcomes in both the training and validation sets. However, the mean absolute error of the validation set (0.082) was higher than that of the training set (0.026). This difference may be related to the smaller validation sample size or variations in data distribution. Therefore, expanding the validation cohort and conducting multicenter studies will be important to confirm the model’s stability and generalizability in broader clinical populations.

This study used multivariate logistic regression with stepwise selection under strict statistical criteria to minimize the effects of confounding factors, thereby enhancing the reliability of variable inclusion. Model performance was assessed in both the training and validation sets, with the latter serving as an independent test for external verification. Although this approach is increasingly common, the validation set AUC of 0.957 in this study demonstrates excellent predictive performance and strong model robustness. Nonetheless, the relatively small validation sample may lead to a slight overestimation of predictive accuracy, underscoring the need for further validation in larger external datasets. In addition, both sensitivity and subgroup analyses were performed to evaluate model stability across different clinical subgroups. The model consistently achieved AUC values above 0.80 across age, prepregnancy BMI, and PCOS subgroups, confirming its broad applicability and robustness in different patient populations. Despite these strengths, several limitations should be acknowledged. First, the retrospective study design may have introduced some degree of selection or information bias. Second, potential confounding variables – such as genetic background, dietary habits, and physical activity – were not included in the current analysis and should be incorporated in future research.^[2,53]^ Finally, since the data were derived from a single-center, the external validity of the findings remains to be verified through large-scale, multicenter prospective studies.

In conclusion, the nomogram developed in this study provides an accurate and clinically useful tool for predicting postpartum MetS risk among women with GDM. It can assist clinicians in identifying high-risk individuals and implementing early, individualized interventions. Future work should integrate additional clinical, genetic, and lifestyle indicators to further enhance the model’s precision and real-world applicability, ultimately improving long-term health outcomes for GDM patients.

## 5. Conclusion

This study established and validated a predictive model for the risk of metabolic syndrome (MetS) in postpartum women with gestational diabetes mellitus (GDM) based on conventional clinical and laboratory indicators. The results showed that PCOS, HOMA-IR, IL-6, HDL-C, and UA were independent risk factors for MetS in GDM patients. The constructed nomogram model demonstrated excellent discrimination and calibration in both the training set and validation set, and showed good robustness and wide applicability in multiple population stratifications and sensitivity analyses. This tool is helpful for clinicians to identify and precisely manage high-risk GDM patients at an early stage, prevent the occurrence of postpartum metabolic complications, and improve maternal and infant health outcomes. Further multicenter, prospective studies are required to enhance and validate the clinical applicability of this model.

## Acknowledgments

The authors thank all the participants and clinical staff at The First Affiliated Hospital of Shandong Second Medical University (Weifang People’s Hospital) for their support in this study. The authors also appreciate the valuable assistance from the laboratory and health management departments involved in data collection and analysis.

## Author contributions

**Conceptualization:** Fei Wang, Yifei Ma, Lixia Cheng.

**Data curation:** Fei Wang, Danyang Ye.

**Formal analysis:** Fei Wang, Danyang Ye.

**Investigation:** Danyang Ye, Yifei Ma.

**Methodology:** Danyang Ye, Yifei Ma, Kai Zhao.

**Resources:** Yifei Ma.

**Supervision:** Zhaofen Liu, Lixia Cheng.

**Validation:** Kai Zhao, Lixia Cheng.

**Visualization:** Kai Zhao.

**Writing – original draft:** Fei Wang, Zhaofen Liu.

**Writing – review & editing:** Lixia Cheng.

## Supplementary Material


