# Supplementary material for: Development and validation of a risk prediction model for postpartum metabolic syndrome in women with gestational diabetes mellitus: A retrospective cohort study
Source: Medicine (Baltimore). 2026 Apr 24;105(17):e48462. doi: 10.1097/MD.0000000000048462 (PMC13124365; doi:10.1097/MD.0000000000048462)
Supplement: Supplementary file 1 [file medi-105-e48462-s001.pdf]

Table S1. Definitions and Reference Ranges of Clinical Variables

No.	Variable Name	Collection Time	Reference Range	Note/Remark
1	Age	First prenatal visit	18–45 years	
2	Pre-BMI	Pre-pregnancy/first visit	18.5–23.9 kg/m²	BMI ≥25: obesity
3	PCOS	Pre-pregnancy/pregnancy	No/Yes	
4	HR	Mid/late pregnancy	60–100 bpm	
5	SBP	Mid/late pregnancy	90–139 mmHg	SBP ≥130: MetS criterion
6	DBP	Mid/late pregnancy	60–89 mmHg	DBP ≥85: MetS criterion
7	GestHyper	Pregnancy	No/Yes	
8	GlyControl	Pregnancy	No/Yes	
9	Asthma	Pregnancy	No/Yes	
10	Dyslipidemia	Pregnancy	No/Yes	
11	Family History of Diabetes	Pregnancy	No/Yes	
12	FBG	24–28 weeks	3.9–5.5 mmol/L	FBG ≥5.6 mmol/L: MetS criterion
13	OGTT-2h	26–28 weeks	<7.8 mmol/L	
14	HbA1c	24–28 weeks	<6.5 %	
15	UA	24–28 weeks	<357 µmol/L	UA ≥357 µmol/L: MetS criterion
16	HOMA-IR	24–28 weeks	<2.5	HOMA-IR ≥2.5: MetS criterion
17	Insulin Index	24–28 weeks	1–6	Formula: Insulin × FBG / 22.5
18	IL-6	24–28 weeks	<5 pg/mL	IL-6 ≥5 pg/mL: MetS criterion
19	Hb	24–28 weeks	110–150 g/L	
20	RDW	24–28 weeks	11–16 %	
21	NLR	24–28 weeks	1–6	
22	PLR	24–28 weeks	90–300	
23	K	24–28 weeks	3.5–5.5 mmol/L	
24	Na	24–28 weeks	135–145 mmol/L	
25	Cl	24–28 weeks	98–107 mmol/L	
26	Ca	24–28 weeks	2.2–2.65 mmol/L	
27	Mg	24–28 weeks	0.75–1.02 mmol/L	
28	TC	24–28 weeks	3.1–5.2 mmol/L	
29	LDL	24–28 weeks	2.07–3.1 mmol/L	
30	TG	24–28 weeks	<1.7 mmol/L	TG ≥1.7 mmol/L: MetS criterion
31	HDL-C	24–28 weeks	1.0–1.8 mmol/L	HDL-C <1.3 mmol/L: MetS criterion
32	NT-proBNP	Late pregnancy	<125 pg/mL	
33	PAP	Late pregnancy	8–20 mmHg	PAP ≥25 mmHg: risk of pulmonary hypertension
34	AFI	Late pregnancy	8–24 cm	
35	EF	Late pregnancy	55–70 %	
36	FVC	Late pregnancy	2.5–4.0 L	
37	FEV1	Late pregnancy	2.0–3.5 L	
38	FEV1/FVC	Late pregnancy	75–80 %	
39	MVV	Late pregnancy	80–120 L/min	
40	Birth Weight	Delivery	2500–4000 g	
41	Neonatal Length	Delivery	48–53 cm	

42	Gest Weeks	Delivery	37–41 weeks
43	NICU Admission	Delivery	No/Yes
44	GDM History	First prenatal visit	No/Yes

Summary of variable definitions, collection times, and reference ranges used in this study. Indicators meeting metabolic syndrome criteria in postpartum GDM women are highlighted, including systolic blood pressure (SBP), diastolic blood pressure (DBP), fasting blood glucose (FBG), triglycerides (TG), high-density lipoprotein cholesterol (HDL-C), homeostasis model assessment of insulin resistance (HOMA-IR), serum uric acid (UA), and interleukin-6 (IL-6).
